# Supplementary material for: Blood pressure and cerebral oxygenation with physiologically-based cord clamping: sub-study of the BabyDUCC trial
Source: Pediatr Res. 2024 Apr 26;96(1):124–31. doi: 10.1038/s41390-024-03131-5 (PMC11257956; doi:10.1038/s41390-024-03131-5)

Supplementary Material

Supplemental Figure 1. The 5th, 10th, 25th, 50th, 75th, 90th, and 95th percentiles for cerebral tissue oxygen saturation ($rStO_2$) for non-randomised infants born vaginally or by caesarean section at ≥35^{+0} weeks' gestation who received deferred cord clamping.

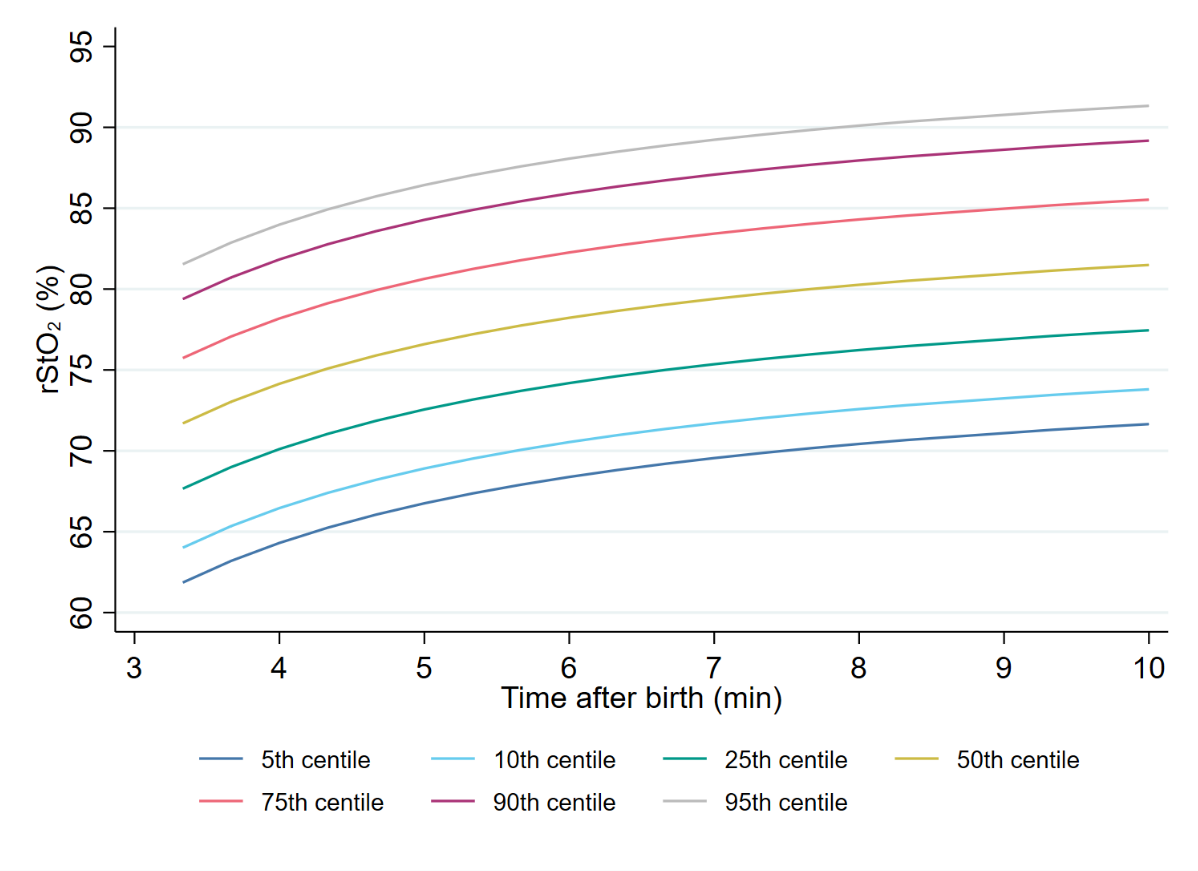

Supplemental Figure 2. Individual trajectories of cerebral tissue oxygen saturation ($rStO_2$) among non-randomised infants born vaginally or by caesarean section at ≥35^{+0} weeks' gestation who received deferred cord clamping.

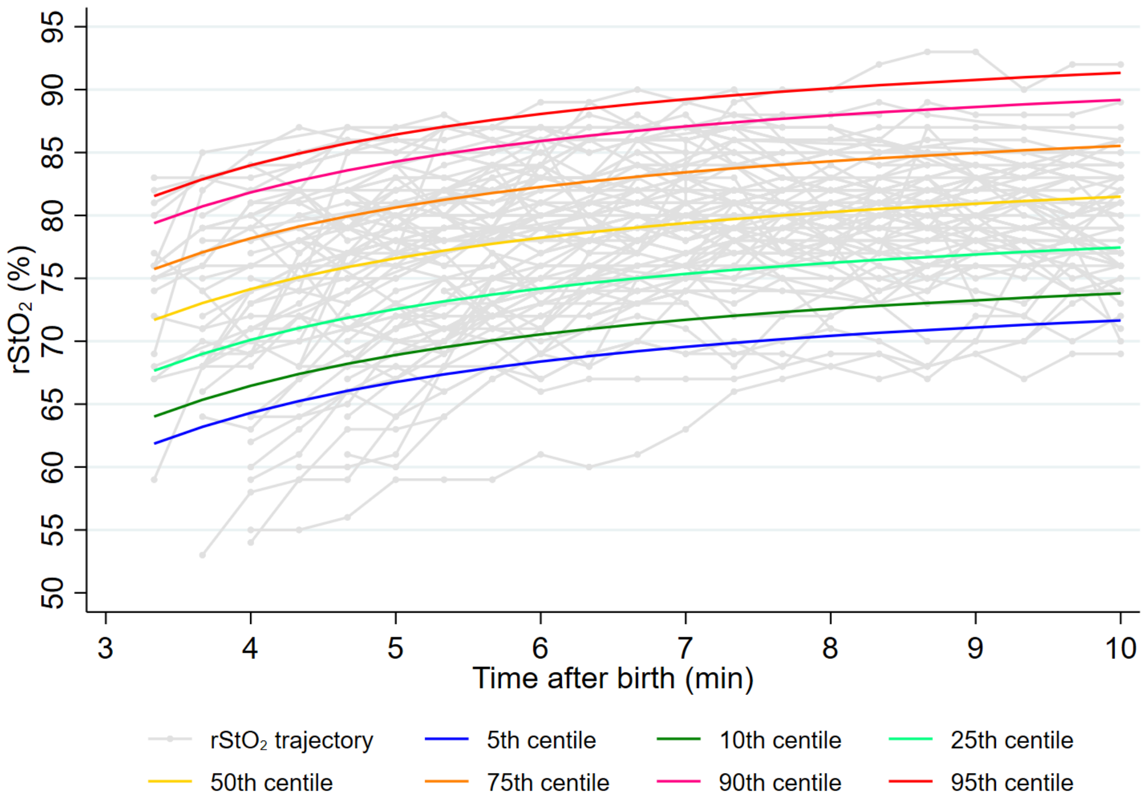

Supplemental Figure 3. The 5th, 10th, 25th, 50th, 75th, 90th, and 95th percentiles for cerebral tissue oxygen saturation ($rStO_2$) for non-randomised infants born by caesarean section (left) and vaginal birth (right) at ≥35^{+0} weeks' gestation who received deferred cord clamping.

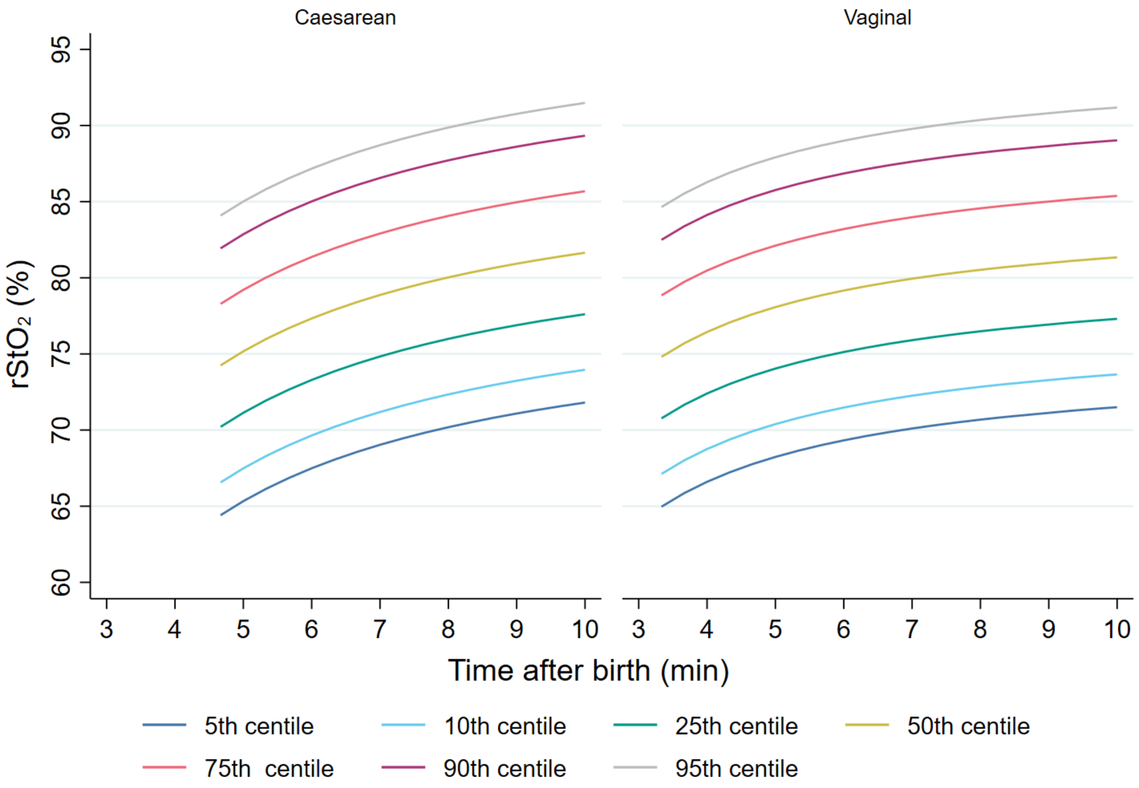

Supplemental Figure 4. The 5th, 10th, 25th, 50th, 75th, 90th, and 95th percentiles for cerebral fractional tissue oxygen extraction (cFTOE) for non-randomised infants born vaginally or by caesarean section at ≥35^{+0} weeks' gestation who received deferred cord clamping.

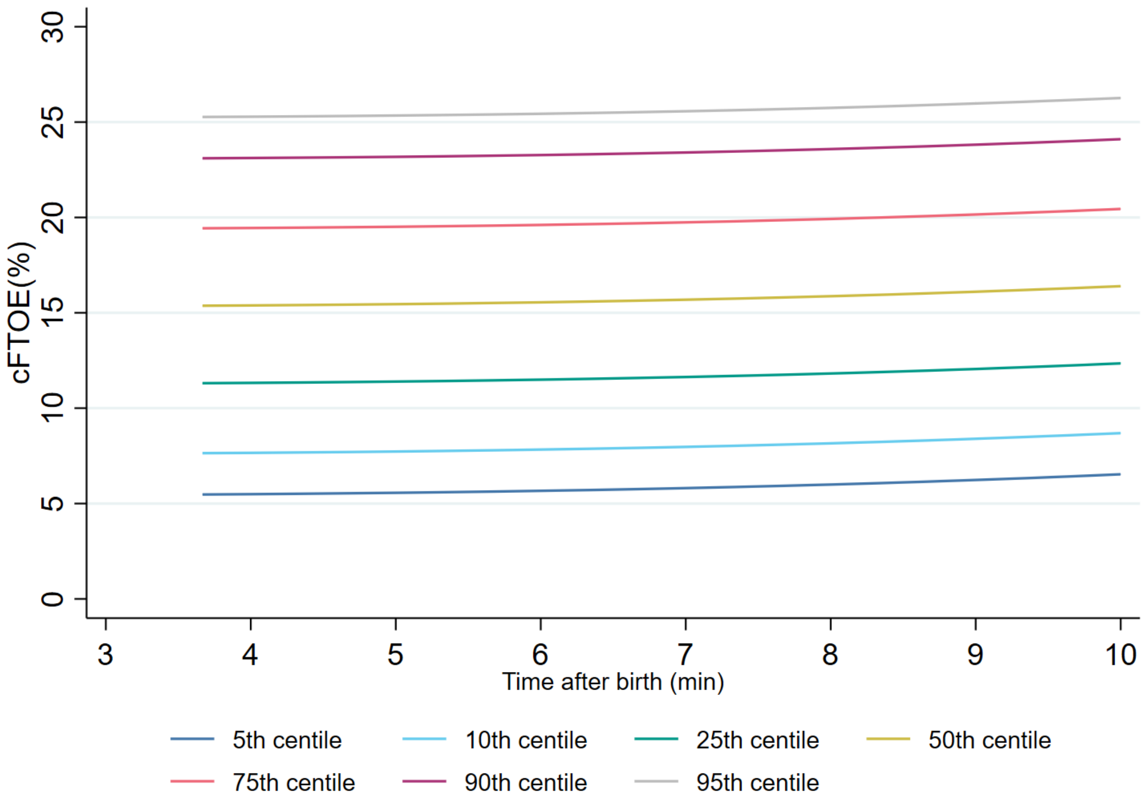

Supplemental Figure 5. Individual trajectories of cerebral fractional tissue oxygen extraction (cFTOE) among non-randomised infants born vaginally or by caesarean section at ≥35^{+0} weeks' gestation who received deferred cord clamping.

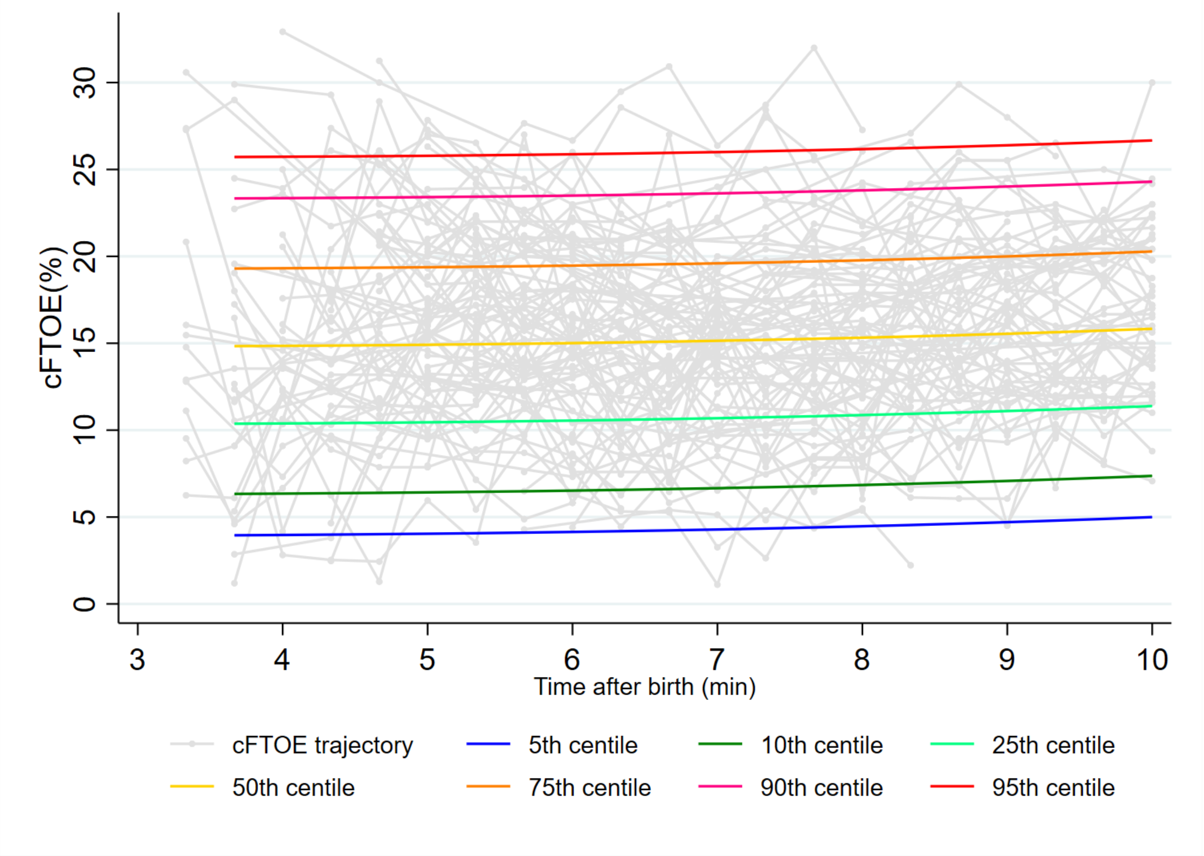

Supplemental Figure 6. The 5th, 10th, 25th, 50th, 75th, 90th, and 95th percentiles for cerebral fractional tissue oxygen extraction (cFTOE) for non-randomised infants born by caesarean section (left) and vaginal birth (right) at ≥35^{+0} weeks' gestation who received deferred cord clamping.

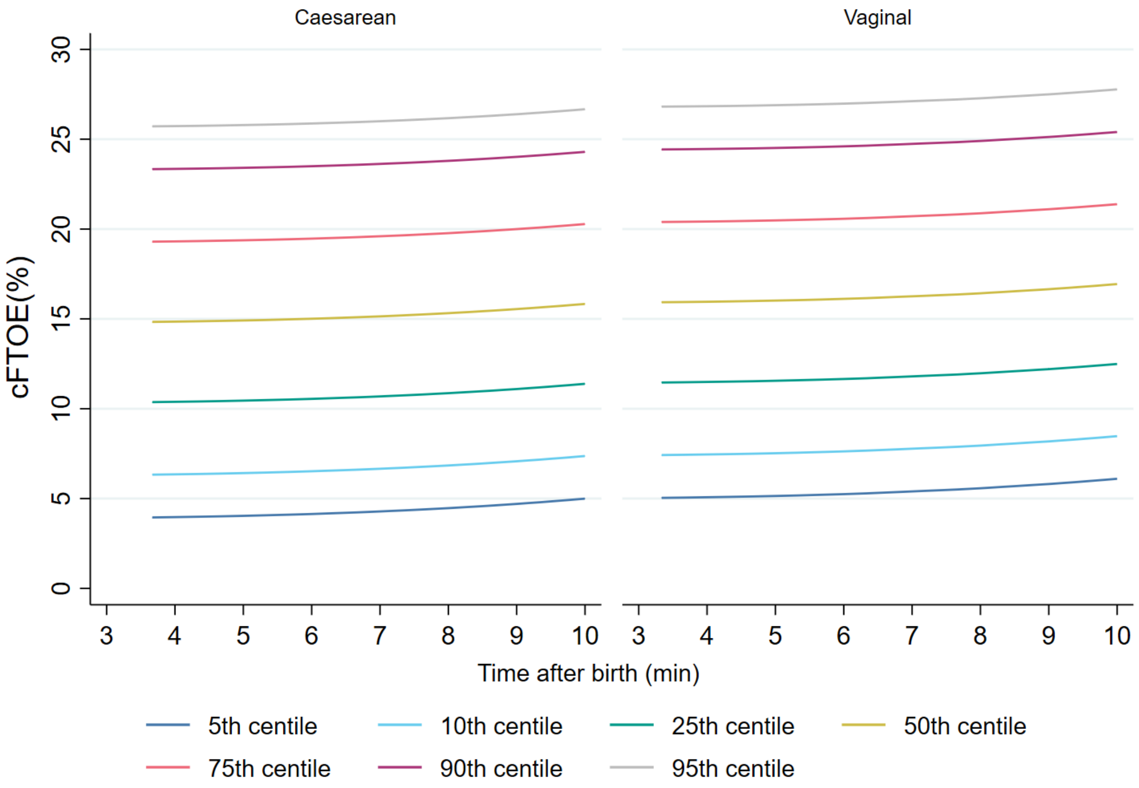

Supplement: Supplementary file 1 — Supplementary Information [file 41390_2024_3131_MOESM1_ESM.pdf]
